# The root apoplastic pH as an integrator of plant signaling

**DOI:** 10.3389/fpls.2022.931979

**Published:** 2022-08-23

**Authors:** Francisco M. Gámez-Arjona, Clara Sánchez-Rodríguez, Juan Carlos Montesinos

**Affiliations:** Department of Biology, ETH Zurich, Zurich, Switzerland

**Keywords:** pH, apoplast, root, signaling, plants

## Abstract

Plant nutrition, growth, and response to environmental stresses are pH-dependent processes that are regulated at the apoplastic and subcellular levels. The root apoplastic pH is especially sensitive to external cues and can also be modified by intracellular inputs, such as hormonal signaling. Optimal crosstalk of the mechanisms involved in the extent and span of the apoplast pH fluctuations promotes plant resilience to detrimental biotic and abiotic factors. The fact that variations in local pHs are a standard mechanism in different signaling pathways indicates that the pH itself can be the pivotal element to provide a physiological context to plant cell regions, allowing a proportional reaction to different situations. This review brings a collective vision of the causes that initiate root apoplastic pHs variations, their interaction, and how they influence root response outcomes.

## Introduction

The concentration of H^+^ (protons) present in all aqueous compartments defines their pH and determines the physicochemical properties of the molecules embedded in the solution. The pH affects both the enzymatic and transporters activity and the protein-protein interactions since the structure and solubility of the proteins are dependent on their net charge and ionization of specific residues (Pace et al., 2009). For this reason, proton levels have a leading role in the development and growth of living organisms. Focusing on plants, pH impacts all essential aspects of their biology such as nutrient absorption by the root, control of the cell wall (CW) expansion, or stomatal movements (Barbez et al., 2017; Geilfus, 2017; Li et al., 2021; Zhang et al., 2021). The pH range found in plant cells is between 4.0 and 8.4, depending on the different subcellular compartments and the rhizosphere (Tsai and Schmidt, 2021). Extreme swings in pH have severe negative repercussions on plant fitness (Ratcliffe, 2003). Consequently, plants possess strong regulatory mechanisms to buffer proton concentration oscillations and to keep the pH within a range compatible with proper growth and development.

**Figure 1:**
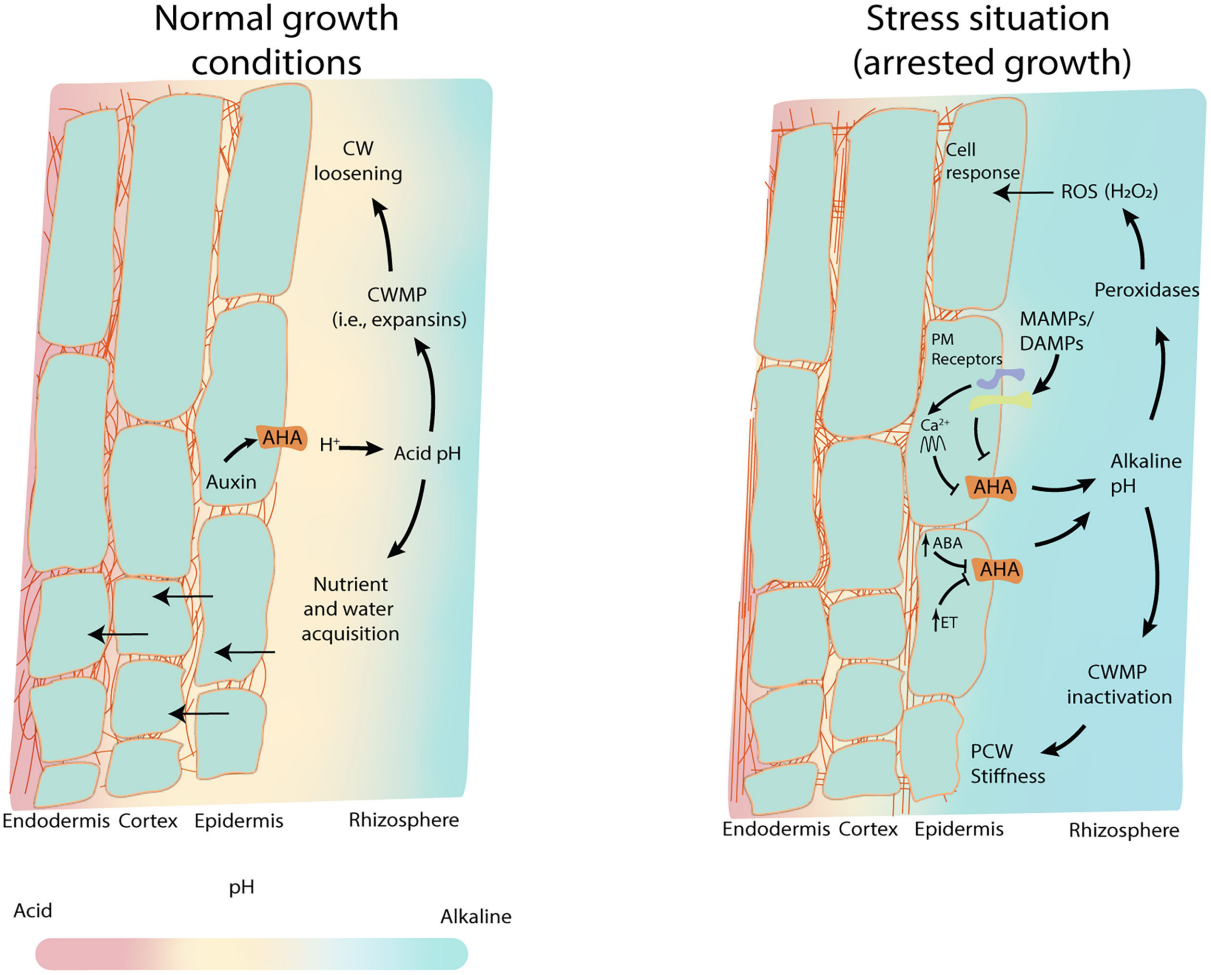
pH_apo_ as the hub for root response. pH_apo_ forms a gradient along the rhizosphere and the different root cell layers, that favors nutrient and water uptake. In normal root growth conditions (left panel), low concentrations of auxin promote plasma proton pumps (AHA) activation lowering the pH_apo_ which induces CW loosening and vacuole regulation for water uptake. This, together with nutrient uptake, boosts cell growth (acid growth theory)_._ Acid pH induces the activation of CW modifying proteins (CWMPs) such as expansins, to promote the CW loosening easing root growth. Under stress situations, hormones such as ABA or ethylene inhibit the AHAs activity fostering the alkalinization of the epidermal pH_apo_ and the rhizosphere. Microbe perception through microbe-associated molecular patterns (MAMPs) and damage-associated molecular pattern (DAMPs) also reduce the activity of AHAs leading to the pH_apo_ alkalinization. In these alkaline conditions, peroxidases generate H_2_O_2_ (Reactive Oxygen Species, ROS), and the CWMPs are inactivated promoting the stiffness of the CW.

Regulation of pH is managed by all subcellular compartments, but it is within the apoplast where the pH (hereafter named pH_apo_) plays a more crucial role in regulating essential processes like intercellular signaling, plant–microbe interactions, plant response to abiotic stresses, and water and nutrient transport (Figure 1) (Monshausen et al., 2009, 2011; Barbez et al., 2017). Plants have different mechanisms to sustain apoplastic H^+^ ions levels in ranges in consonance with the plant physiology. This includes plasma membrane proton pumps, organic anion release, root respiration, redox-coupled process (Hinsinger et al., 2003), and the buffer capacity of the plant CW (Martinière et al., 2018). Although they do not act directly in buffering pH, phytohormones have a strong impact on the regulation of pH_apo_. While the role of auxin or abscisic acid (ABA) in pH_apo_ has broadly been studied, other hormones are getting more attention as modulators of proton pump activity, such as cytokinin (Falhof et al., 2016).

Environmental challenges such as high salinity, drought, anoxia, or microbes induce drastic pH_apo_ changes provoking a systemic plant response (Felle et al., 2005; Minibayeva et al., 2015; Geilfus, 2017; Geilfus et al., 2017; Kesten et al., 2019). A specific example of this influence happens during high rainfalls. The dissolved atmospheric carbon dioxide (CO_2_) forms carbonic acid (H_2_CO_3_), which together with the atmospheric pollutants acidify the soil (Blake, 2005). This low external pH increases the availability of Mn^2+^ and/or Al^3+^ inhibiting plant growth (Msimbira and Smith, 2020). Interestingly, in some cases, the same stress induces different variations in the pH_apo_. The plant pathogen *Fusarium oxysporum* induces a fast root pH_apo_ acidification during its first contact with the root, while the pH_apo_ is alkalinized in later stages of the infection (Kesten et al., 2019). In this review, we revisit the role of the root pH_apo_ in cellular signaling and how it is regulated. Root pH_apo_ values are especially dynamic, changing more than two pH units upon certain stresses (Geilfus, 2017), likely because molecules diffusion between the root apoplast and the exterior is happening with high permeability. Additionally, the pH_apo_ change depends on the intensity of the stress. For example, pH_apo_ increases proportionally to the concentration of NaCl (Geilfus and Mühling, 2012). Importantly, as with other signaling mechanisms, the pH_apo_ values tend to return to their steady state after their modification.

The acid growth theory is broadly assumed in the plant scientific community, which affirms that while acid pH_apo_ enables cell growth, alkaline pH_apo_ induces CW stiffness and protection against osmotic stress and pathogens ultimately arresting cell growth (Takahashi et al., 2012; Barbez et al., 2017; Du et al., 2020). The fact that so many different processes are linked to the pH_apo_ brings to light the pivotal role of the H^+^ levels in the development of the plant. We consider that the root pH_apo_ acts as a regulatory element, which uses the local levels of protons as a link between the external environment and plant physiology. The oscillation of the pH_apo_ during different stresses would be a way through which plant development systems coordinate to react properly. Furthermore, pH levels coordinate with cytosolic secondary messengers such as Ca^2+^, allowing the translation and scalability of the external signalization to the cytosol (Behera et al., 2018). We present here, an overview of the processes that can modulate the root pH_apo_, how the rhizosphere and the phytohormones participate in this process, and how the connection among these elements determines root response (growth and nutrient absorption).

## pH_apo_ in the root and rhizosphere

There are different methods to estimate the pH of the plant cell and its subcellular compartments, including the apoplast. The most used and accurate methods are based on the use of dyes (such as Bromocresol) *in vivo* pH-sensor based of fluorescent probes, H^+^-selective microelectrodes, or ^31^P nuclear magnetic resonance spectroscopy [reviewed in Geilfus (2017) and Tsai and Schmidt (2021)]. Thanks to these approaches, we know that the pH is not constant across the length and tissues of the root and additionally varies within the different subcellular compartments of the cell. Concretely, the root pH_apo_ oscillates in an acidic range between 4.5 and 5.5, assuming normal growth conditions (Felle, 2001; Tsai and Schmidt, 2021). Ultimately, this allows for cell elongation. In contrast, the pH in the cytosol is slightly alkaline, with values around 7.5 (Felle, 2001). The cytosol has a stronger buffer capacity (20 to 80 mM H^+^ per pH unit) than the apoplast (low millimolar range per pH unit) (Oja et al., 1999; Felle, 2001), probably because dramatic pH changes within the cytosol would likely produce devastating effects on the major part of the cellular metabolism processes, small pH changes are enough to act as the signaling element (Kader and Lindberg, 2010). Thus, pH_apo_ has a higher dynamic range than the cytosolic pH, which potentially might give the apoplast the capacity to create more complex signaling. The root apoplast is a continuum through different cell root layers. However, the pH_apo_ of the endodermis and the stele are significantly more acidic than the pH_apo_ of the root external cell layers in mature areas of the root (Martinière et al., 2018). Plants keep this radial variation in pH even when the culture media surrounding the plant is significantly more alkaline (Martinière et al., 2018). One possible explanation that could justify the differences in the pH_apo_ along the radial root cell layers is the establishment of a pH gradient that allows for the directionality of the nutrients acquisition. However, it is unknown whether the pH_apo_ of the inner layers of the root becomes more alkaline upon stress, as it happens within the epidermal cell upon treatment with microbe-associated molecular pattern such as chitin (Felle et al., 2009; Kesten et al., 2019).

The rhizosphere is the volume of soil that has a direct exchange of molecules with the root. The nature of the root-soil interphase is influenced by the root secretions, the soil composition, and their associated microorganisms. There are substantial levels of molecule diffusion between the rhizosphere and the apoplast of the root outer layers which hints at the strong influence of the rhizosphere over the root pH_apo_ (Alassimone et al., 2012). Interestingly, the pH_apo_ close to the plasma membrane (PM) stays acid even when the pH of the media gets significantly more alkaline, highlighting the strong regulatory mechanisms involved in maintaining the pH stable (Martinière et al., 2018). The rhizosphere pH is an essential element in plant nutrient acquisition because it determines the solubility and availability of these nutrients. In general, plants are adapted to grow in soils with a pH in the range between 5.5 and 7.5 because in these conditions the nutrient availability is optimized (Msimbira and Smith, 2020) (Figure 1). Acid soils favor micronutrients such as iron (Fe) or manganese (Mn) acquisition, while neutral or slightly alkaline soils increase the availability of macronutrients such as nitrogen (N), phosphorous (P), or potassium (K). Nevertheless, the rhizosphere pH must be balanced to avoid the deficiency or toxicity, respectively, of some ions such as Al^+3^ or Fe^2+^ (Msimbira and Smith, 2020). Hence, plants have different mechanisms to actively change the pH_apo_, which will also influence the rhizosphere's pH. In fact, it has been observed that the overexpression of H^+^-ATPase in rice plants changes the rhizosphere's pH, enhances nutrient uptake, and increases yield production (Zhang et al., 2021). Thus, the modulation of root pH_apo_ represents an interesting target in biotechnology.

## Plant mechanisms to modify the root pH_apo_

**Figure 2:**
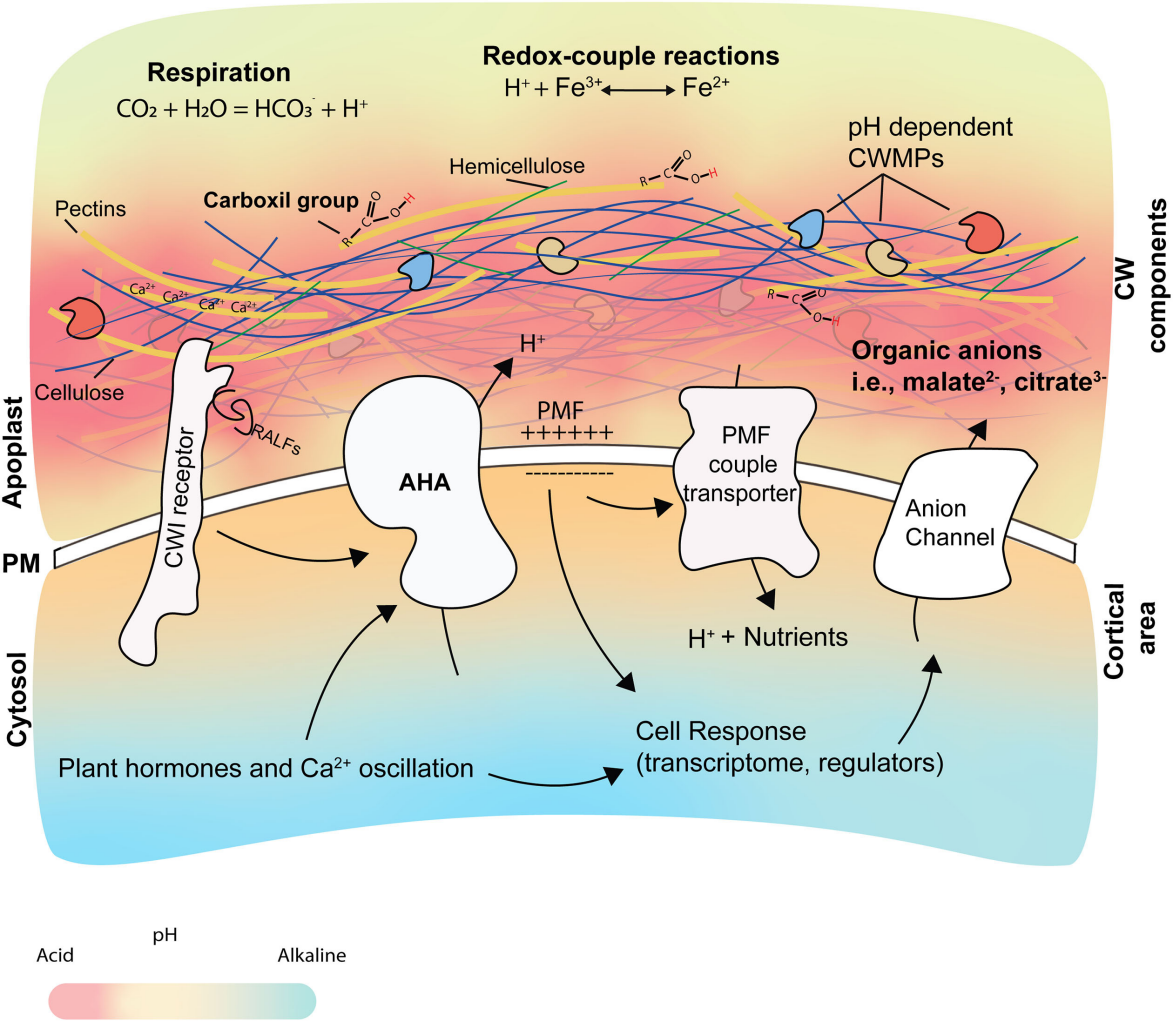
Origin and consequences of pH_apo_ changes. The root pH_apo_ is determined by different cellular elements and processes, such as root respiration, redox-coupled reactions, CW structure, organic acids, and the activity of AHAs. Changes in pH_apo_ affect the activity of CW modifying proteins (CWMPs), water and nutrient uptake, and the cellular response. The pKa is predicted to be more acidic in the CW than the apoplastic fluid. The AHA activity participates in the creation of the proton motive force (PMF), which is used by different transporters to internalize nutrients. Calcium ions (showed as Ca^2+^) contribute to CW structure by interacting with pectins and forming the egg- box in a pH-dependent manner. AHA activity is regulated by phytohormones and by CW integrity sensors after perceiving signals at the apoplast (like RALFs and DAMPs), leading to apoplast alkalization.

The root pH_apo_ is altered through endogenous and exogenous processes. Here, we will focus on the endogenous, plant-mediated processes which play a dominant role in the establishment of the pH_apo_, i.e., PM proton pumps activity, root respiration, organic anion release, redox-coupled process, and the CW (Figure 2).

### Plasma membrane proton pumps

Extrusion of H^+^ by the proton pumps at the PM is the dominant mechanism used by plants to regulate the differential pH between the cytosol and the apoplast (Figures 1, 2). These pumps use the energy derived by the ATP to create a proton gradient that contributes to the generation of a proton motive force (PMF) (Haruta and Sussman, 2012; Wegner and Shabala, 2020). This PMF is used to then drive solute and water uptake into the cell (Cosse and Seidel, 2021). Among the 11 plasma proton pumps in *Arabidopsis thaliana*, named AUTOINHIBITED H^+^-ATPases (AHAs) (Axelsen and Palmgren, 2001), the most expressed ones are AHA1 and AHA2, that share an elevated level of functional complementation, and which importance is evidenced by the embryo lethality of the double mutant (Haruta et al., 2010). Importantly, the fact that the overexpression of AHAs does not show any obvious phenotypical effect suggests that the levels of these proteins are tightly controlled and/or the regulation of their activity is mainly acting posttranslationaly. AHA activity is regulated through the phosphorylation/de-phosphorylation of different amino acid residues by various proteinaceous interaction partners. These interactions are driven by phytohormones, lipids, and Ca^2+^ signaling, all of which are highly conserved among plants (Haruta et al., 2015; Falhof et al., 2016). This plethora of AHAs-activity regulators shows that these pumps have an extraordinary impact on many key aspects of plant biology and any disturbance could have disastrous consequences for plant fitness (Gévaudant et al., 2007). However, the identity of direct regulators of AHAs is still elusive. It is also unknown and unexplored whether the variations in the level of activation of the AHAs are part of a signaling cascade on their own, in addition to the role of the pH as a secondary messenger. Indeed, mutants displaying AHA overactivation, such as the mutant on the companion cellulose synthase (*cc1cc2*) or FERONIA (*fer-4*), show an upregulation of defense-related genes, making them more tolerant to pathogen attack and supporting the potential direct connection between AHA activity and plant defense (Masachis et al., 2016; Kesten et al., 2019).

### Root respiration

Root respiration produces at least 50% of the CO_2_ present in the soil (Pregitzer et al., 2007). When there is high availability of N in the soil, plants can enhance their growth rate, increasing significantly the CO_2_ concentration in these soils (Pregitzer et al., 1998). The CO_2_ present in the soil interacts with the water forming weak acid carbonate (H_2_CO_3_) that, in neutral and alkaline environments, will release H^+^ and acidify the apoplast and the rhizosphere (Figure 2).

### Organic anions

Malate, oxalate, fumarate, malonate, succinate, and oxalacetate are some of the organic anions found in root exudates (Vančura and Hovadík, 1965; Wegner et al., 2021). They are released by the cell in a deprotonated form because their pKa is usually lower than the pH_apo_ (Hinsinger et al., 2003), helping the cell to buffer the proton extrusion generated by the AHAs when the pH_apo_ becomes too acidic (Figure 2). For this reason, some of the organic anions transporters at the PM are activated upon extracellular pH drop (Liang et al., 2013). Moreover, these molecules are secreted by the roots to improve P acquisition (Lambers et al., 2006) or increase plant tolerance to elevated levels of aluminum (Al) (Yang et al., 2013), which might take place during rhizosphere acidification.

### Redox-coupled process

The oxidation state of key elements such as Fe, Mn, N, or Sulfur (S) is coupled with the pH_apo_ because the direction of the reaction to change their oxidation state is conditioned by the amount of H^+^ present in the media (Hinsinger et al., 2003). For example, the chemical reduction of Fe^3+^ consumes H^+^ ions increasing the pH_apo_, while the chemical oxidation of Fe^2+^ acidifies the soil (van Breemen, 1987) (Figure 2).

### The cell wall

The plant CW is mainly composed of the carbohydrates cellulose, hemicellulose, and pectin, with proteins embedded in it. Pectin is enriched in uronic acids, mostly galacturonic acids (GalAs), whose carboxyl group is negatively charged. Pectin is secreted to the apoplast and heavily methylated at their GalAs. Its demethylation at the CW and the potential link with Ca^2+^ ions to form the so-called egg-boxes alters the charge balance of the CW (Shomer et al., 2003). The pKa value of carboxyl groups in the GalAs is between 4.0 and 5.0, which means they behave as weak acids helping to maintain acidic apoplastic spaces (Meychik and Yermakov, 2001). The CW arabinogalactan proteins also contain uronic acids (Seifert and Roberts, 2007) which, potentially, could also contribute to the CW charge, although the quantitative importance of this possibility is not clear. It has been reported that the pH of the CW is more acid than the apoplastic fluid (Sentenac and Grignon, 1981, 1987), suggesting that the CW carboxyl groups might be highly protonated, based on their pKa, in a steady state apoplast (Figure 2), but this should be corroborated with more accurate methods.

All these mechanisms are linked to environmental conditions. Proton pumps are induced in Fe-deficient roots (Santi and Schmidt, 2009), and their overexpression enhances N and C uptake by rice (Zhang et al., 2021). Redox-coupled reactions with Fe, N, or S might cause temporal variation in the soil pH during seasonal flooding (van Breemen, 1987). Organic acids produce changes in soil pH increasing nutrient availability (Liu et al., 2022) and might contribute to microbial growth, which together with root respiration, will produce CO_2_ with a strong influence on plant biology in alkaline soils (Hinsinger et al., 2003). Overall, the plant mechanisms to modify pH_apo_ have a critical role under stress conditions, such as high salinity or drought (Miao et al., 2022).

## Plants hormones and pH_apo_

Phytohormone signaling pathways can be triggered in response to different stresses and help the plant to adjust to specific necessities. Some of the hormonal signaling pathways alter the pH_apo_ as a consequence of their response mechanisms.

Abscisic acid (ABA) is considered a stress-response hormone whose signaling is triggered as a result of environmental stress including drought, high soil salinity, temperature, and metal soil contamination (Roberts and Snowman, 2000; Zörb et al., 2013; Urano et al., 2017). Treatment with ABA specifically inhibits the AHA activity, alkalizing the apoplast and impairing the hypocotyl elongation (Hayashi et al., 2014) or root growth (Planes et al., 2015). However, the downregulation of AHA activity induced by ABA in guard cells is determinant of stomatal closure (Bauer et al., 2013; Miao et al., 2022). Inversely, lower concentrations of ABA activate the AHAs' activity, stimulating the root growth (Miao et al., 2021). Certainly, there is controversy among published ABA data regarding its influence on pH_apo_, pointing out that the effect of ABA on pH_apo_ might be concentration- and cell-type-dependent. ABA also exerts different effects on different plant species; for example, saline stress and ABA-mediated response cause the inhibition of the activity of proton pumps and pH_apo_ alkalinization in tomato roots (Gronwald et al., 1990), whilst in cucumber roots, the same conditions activate the activity of the proton pumps (Janicka-Russak and Kłobus, 2007). The different effects that ABA has on the pH_apo_ suggest that it is not the only factor influencing AHA activity and other signaling elements seem to be essential in regulating the pH_apo._

Over the last years, it has been extensively discussed the role of auxin in plant cell growth. Auxin enhances AHA activity by mediating the phosphorylation of the penultimate threonine residue within the C-terminal autoinhibitory domain of AHAs (Takahashi et al., 2012). This auxin's AHA activation implies the induction of *SAUR19* gene expression, which inhibits the PP2C-D phosphatase activity, required for the dephosphorylation of the C-terminal autoinhibitory domain of AHAs. Since PP2C-D is not active, the AHA's C-terminal autoinhibitory domain remains active (Spartz et al., 2014). The activation of AHAs allows for the loosening of the CW (read below, section the impact of pHapo on apoplastic biology) facilitating cell growth (Barbez et al., 2017; Du et al., 2020). The role of auxin in regulating the pH_apo_ is biphasic and dependent on its concentration. The above mechanism describing AHA activation and the pH_apo_ acidification by auxin is observed under endogenous auxin concentrations. However, high levels of auxin applied externally trigger a fast pH_apo_ alkalinization that requires the FERONIA receptor activity and induces the concomitant inhibition of cell expansion (Barbez et al., 2017). Thus, a controversial aspect of the auxin effect on the pH_apo_ has raised in the last years, where the auxin regulation of the pH_apo_ seems to be dependent not only on its local concentration but also on the cell type or/and plant tissue where auxin acts. It has recently been reported that the same auxin concentration can promote growth in shoots while having the opposite effect on roots. This implies the existence of differential activity of auxin receptors, such as TIR1/AFB and TMK1, that is dependent on tissue localization (Li et al., 2021; Lin et al., 2021).

The function and signaling of the gaseous hormone ethylene (ET) are intimately related to auxin in regulating plant development. ET is also a major regulator of stress responses (Vaseva et al., 2018), and the external application of ET or its precursor, the 1-aminocyclopropane-1-carboxylic acid, induces a fast apoplastic alkalization inhibiting the fast cell elongation of epidermal root cells (Staal et al., 2011). ET modulates the alkaline stress-mediated inhibition of root growth by increasing auxin accumulation via induction of auxin biosynthesis-related genes, control of local auxin biosynthesis, and the expression of the auxin transporter AUX1 (Li et al., 2015; Vaseva et al., 2018) (Figure 2). ET signaling can also modulate cellular responses involved in plant acclimation to acidic pH by regulating the activity of the class III peroxidases (CIII Prxs). The CIII-Prx produces CW modifications, such as hydroxyproline-rich glycoproteins crosslinking and callose deposition lead to higher CW stiffness, and higher tolerance to extremely low pH ultimately leads to an arrest in cell expansion during stress conditions (De Cnodder et al., 2005; Graças et al., 2021).

Cytokinin (CK) hormonal actions usually antagonize those of auxin effects and can counterpart its signaling. CK has a key role in root growth, intervening in the balance between cell division and cell differentiation within the root tip (Dello Ioio et al., 2007; Montesinos et al., 2020). CK participates in the establishment of the root transition zone, determining the root meristem size, through the activation of the AHAs that acidify the pH_apo_ and the activation of the α-expansin EXPA1. The latter helps in the loosening of the CW, and the beginning of the cell expansion in the root elongation zone. Thus, the pH_apo_ acidification of the root mediated by the CK activation of AHA1 and AHA2 proton pumps is necessary to control the initiation of cell differentiation (Pacifici et al., 2018). Curiously, although auxin and CK usually have opposing effects, they can promote similar cellular responses that are dependent on their concentration, signal duration, and cell type.

Brassinosteroids (BRs) are plant steroidal hormones that, similar to auxin and CK, participate in the regulation of plant growth and differentiation by controlling cell division and expansion. BRs can activate the AHAs during hypocotyl elongation by inducing the phosphorylation in their penultimate amino acid (Thr) (Minami et al., 2019). In this line, it was shown that the BR-receptor BRI1-BAK1 system interacts directly with AHA2 and AHA7 proteins *in vivo*. However, it remains unclear whether this interaction is enough to phosphorylate and activate these H^+^-ATPases (Miao et al., 2018, 2022; Yuan et al., 2018). Remarkably, little is known about how BRs can modify the pH_apo_ in root cells and further investigation in this direction will be necessary for the coming years.

The fact that different phytohormones converge in regulating the pH_apo_ supports the idea that pH_apo_ might be a universal language among cells to coordinate cell responses.

Another plant's short-distance communication signaling mechanism is mediated by the RALF (Rapid Alkalinization Factor) small signaling peptides (Figure 2). RALFs peptides regulate plant patterning and development (Murphy and De Smet, 2014). This family of small peptides holds 34 members in *A. thaliana*, and as its name indicates, they have been associated with alkalinization of the extracellular medium (Pearce et al., 2001; Blackburn et al., 2020). Concretely, RALF1 interacts with the PM-localized receptor FERONIA (FER) to regulate the pH_apo_. This interaction leads to AHA2 phosphorylation reducing its activity and a fast alkalinization of the apoplast that inhibits root cell elongation (Haruta et al., 2014). RALF33 and RALFL36 treatment decreased by 3-fold the H^+^ pumping activity of H^+^-ATPases and increase the pH_apo_, inhibiting root growth (Gjetting et al., 2020). RALF23 overexpression leads to an alkalinization of the rhizosphere and inhibition of the root growth (Srivastava et al., 2009). RALF23 could exert part of its function by altering the nanoscale organization of the BRs-receptor BAK1, and its interaction with FER (Gronnier et al., 2022). Interestingly, pathogens like *Fusarium oxysporum* also secrete RALF-like peptides during their plant infection to produce a pH_apo_ alkalinization that enables fungal colonization (Masachis et al., 2016).

Overall, having such a complex and evolving system that includes hormonal and small peptides regulation to modify pH_apo_ suggests that pH_apo_ is a way to escalate the cell response signal, acting as an element to regulate the activity and properties of many different proteins and molecules at once.

## The impact of pH_apo_ on apoplastic biology

### Cell wall remodeling

Changes in pH_apo_ have a direct impact on the CW integrity and the properties of the proteins embedded in it. The balance of protonation in pectin carboxyl groups determines their capacity to interact with cations, mostly Ca^2+^ to form the egg-box, and affects the pectin–cellulose and pectin–pectin interactions, which has a significant impact on the structure of the CW (Meychik et al., 2021) (Figure 2). During plant cell elongation, precise and local acidification cycles of pH changes occur, changing the CW components' interaction and causing a CW-restructuring without compromising the cell integrity leads to unidirectional cell growth (Hocq et al., 2017; Arsuffi and Braybrook, 2018; Majda and Robert, 2018). The activity of several CW modifying proteins (CWMPs), such as expansins, is pH-dependent (Cosgrove, 1999; Sampedro and Cosgrove, 2005). The precise control of H^+^ fluxes and the spatio-temporal specificity of expansins expression and localization are essential in cell elongation (Samalova et al., 2022). In fact, salt stress alters the expansins expression pattern in wheat, affecting the cell wall extensibility under different pH_apo_ (Shao et al., 2021). Expansins promote the loosening of the CW while the increase of the turgor pressure created by the enhanced intake of K^+^ allows the cells to grow (Channels, 1991; Mathur and Hülskamp, 2001; Hager, 2003; Velasquez et al., 2016; Phyo et al., 2019). On the other hand, alkaline pHs induce the activity of other CWMPs, such as extensins and peroxidases, which fortify the CW (Castilleux et al., 2021). Overall, we propose that the ability to create microdomains within the apoplast, by tightly regulating the pH_apo_ oscillations, might allow the cell to precisely modify its CW and adapt its growth to its developmental needs. However, this idea needs further experimental validation.

### Apoplastic ion balance

The homeostatic balance of certain ions between the apoplast and the cytosol is a key element in plant development and is closely related to proton level regulation. The strong ion difference (SID) represents the net charge between the cations and anions and it has a direct influence on the pH (Gerendás and Schurr, 1999). Proton ATPases energize ions secondary transporters, such as K^+^, Mg^2+^, or Cl^−^, to balance the net charge created by proton transport and to permit a pH gradient (Good, 1988; Hangarter and Good, 1988; Gerendás and Schurr, 1999). K^+^ is one of the most abundant cations present in plants since it is essential for their growth, and it has a strong connection with the pH_apo_. In a situation with low levels of apoplastic K^+^, acidification of the extracellular space enhances K^+^ uptake, allowing for cell growth (Chen and Gabelman, 2000; Minjian et al., 2007). On other occasions, the pH_apo_ is affected by high levels of toxic anions, such as Na^+^ which induces apoplastic alkalinization (Foster and Miklavcic, 2020) or NH4^+^ which produces apoplastic acidification (Liu and von Wirén, 2017). Likely, it is in those moments, in which the apoplastic balance ion is affected, when the pH_apo_ might act as a signal to activate the cell response.

The CW also takes part in the apoplastic cation/anion balance in a pH-dependent manner. In standard growth conditions, pectin binds Ca^2+^ and B^3+^ (O'Neill et al., 2004; Phyo et al., 2019). However, those cations might be displaced by protons if the pH decreases significantly thus modifying the CW structure. Moreover, in the case of a significant increase for another cation, such as Na^+^ during saline stress, we hypothesize that Na^+^ could compete for the negative charges at the CW, and replace protons, boron, and calcium from the carboxyl groups of pectins and AGPs. In fact, during salt stress the apoplastic space becomes alkaline which might enhance the interaction of Na^+^ with the CW, allowing its compartmentalization and decreasing its toxicity. Interestingly, treatment with anions like SO${}_{4}^{2-}$ or NO${}_{3}^{-}$, which are abundant in nature, does not trigger apoplastic pH changes, suggesting a strong compensatory mechanism related to the presence of anions (Martinière et al., 2018).

### Plasma membrane transporters' activity

The activity of several transporters is connected with the H^+^ flux across the PM (Zhou et al., 2021). In Arabidopsis, pH 6.6 seems to be optimal for AHA transport activity (Olivari et al., 1993; Hoffmann et al., 2019), although, at lower pHs, the activity of the AHAs is enhanced due to the cytosolic acidification (Liang et al., 2020). The Na^+^/H^+^ exchangers (NHXs) use the H^+^ influx to pump Na^+^ extracellularly (Aharon et al., 2003), and their activity might be enhanced with higher AHA activity (Fan et al., 2019). This leads us to think that the apoplastic alkalinization observed upon salt stress might facilitate AHA's activity. The cation/H^+^ exchangers (CHX) are hypothesized to use the H^+^ gradient in a similar way to that described for NHXs, but it needs to be experimentally demonstrated (Sze and Chanroj, 2018). The extracellular acidification also stimulates the activity of N transporters such as ammonium transporter (AMT) or nitrate transporter (NRT) (Søgaard et al., 2009; Fan et al., 2016). The activity of the different isoforms of phosphate transporters (PHT) has different optimal pHs (Ai et al., 2009; Sun et al., 2012; Wang et al., 2014). Interestingly, the loss of AHA2 function coincides with the downregulation of K transporters (Hoffmann et al., 2019). All these examples underline that the pH_apo_ influences many of the most relevant families of ion transporters with significant consequences in plant physiology.

### Intracellular communication: Electric signaling, calcium, glutamate, and ROS

Plants need to adapt constantly to a dynamic environment, and for that, they require systems that monitor multiple stimuli and integrate this information to generate long-distance signals that serve for communication and generation of responses in different tissues and organs. The unification of these different cellular signaling systems has been an object of study in the last decades. The pH_apo_ is closely related to electric signaling, glutamate, Ca^2+^, and ROS systems, interacting with each other to provide the appropriate duration, localization, and physiological context in response to the stimuli that generate the signals (Johns et al., 2021). Different examples illustrate how pH_apo_ coordinates with Ca^2+^ and the generated electrical signals, also named slow wave potentials (SWPs). For example, the inactivation of the electrogenic proton pumps, and consequent alkalinization of the pH_apo_, have been implicated in the generation of SWPs (Kumari et al., 2019); or the constitutive activation of AHA1 inhibits both Ca^2+^ waves and SWP generation (Shao et al., 2020). Furthermore, the reactivation of the AHA1 activity is fundamental for the repolarization of the PM and the restoration of the membrane potential after the signal transmission (Kumari et al., 2019; Johns et al., 2021).

Ion channels like the GLUTAMATE RECEPTOR–LIKE (GLR) family act as sensors that convert the external signal into an increase in intracellular Ca^2+^ concentration, generating Ca^2+^ waves and the SWPs, that propagate to distant organs (Toyota et al., 2018). The fact that the GLRs (GLR3.3 and GLR3.6) are only active when the pH_apo_ is above 6.5, indicates that alkaline pH_apo_ is an essential condition for these channels for opening (Shao et al., 2020). Furthermore, the constant activation of H^+^-ATPases by fusicoccin generates an increase in the extracellular protons, which impairs the depolarization phase of glutamate-induced SWPs (Shao et al., 2020), illustrating once more, the high dependency on the activity of these transporters on pH_apo_. Other calcium transporters such as CNGC14 also show the existing connection between Ca^2+^ signaling and pH_apo_, since the loss-of-function *cngc14* mutant losses the capacity to alkalinize the apoplast after high auxin concentration treatments (Shih et al., 2015). In general, different abiotic stresses produce different hallmarks in pH_apo_ and Ca^2+^ dynamics in the cytosol and the apoplast, this variety of signaling signatures allows the cell to respond accordingly to the specific stimulus (Gao et al., 2004).

Reactive oxygen species (ROS) waves can propagate through different plant tissues similarly to electric and calcium waves during long-distance signaling (Johns et al., 2021). The increased levels of intracellular Ca^2+^ can activate the RESPIRATORY BURST OXIDASE HOMOLOG D (RBOHD) PM-localized enzymes, that synthesize ROS, such as H_2_O_2_, in the apoplastic space during the plant electrical/ionic system signaling (Gilroy et al., 2016; Johns et al., 2021). Likewise, pH_apo_ affects ROS production, being the alkalization of the apoplast an essential step for the generation of H_2_O_2_ (Bolwell et al., 1995; Monshausen et al., 2007). The PM is permeant to H_2_O_2_ and thereby variations in pH_apo_ can be translated to the interior of the cell. The presence of H_2_O_2_ will amplify the signaling by interacting with proteins with redox-sensitive moieties in the cytosol or other subcellular compartments (Antunes and Brito, 2017; Rampon et al., 2018; Janku et al., 2019). Moreover, oscillations in cytosolic H_2_O_2_ might modulate the AHA activity and participate in pH_apo_ regulation (Mangano et al., 2018). While pH_apo_ alkalinization contributes to CW stiffening through H_2_O_2_ production, low pH_apo_ levels can help the protonation of superoxide anion radicals (i.e., •OH), which promote CW loosening (Pottosin et al., 2014). Contrary to what one might think, active oxygen species production does not seem to contribute to alkalinization of the apoplast by H^+^ consumption, since in tobacco cells have been observed that the NtrbohD oxidase activity does not affect the extracellular pH (Simon-Plas et al., 2002).

## Root pH_apo_ in stress situations

### Abiotic stress

Environmental events affect simultaneously the rhizosphere and the pH_apo_. Some of them promote acidification, like high rainfall, ammonium-based fertilizers, and plant growth on its own (Msimbira and Smith, 2020). On the other hand, irrigation with water containing prominent levels of bicarbonates or drought tends to alkalinize the rhizosphere in a long term (Odutola Oshunsanya, 2019). Saline stress (NaCl) is one of the most common severe stresses faced by plants, producing pH_apo_ alkalinization because of two events happening concomitantly: Cl^−^ ions are internalized via symport with 2 H^+^ using the proton gradient generated by the AHAs (Geilfus, 2017) and salt-induced ABA-increase downregulates the AHA activity (Falhof et al., 2016; Geilfus, 2017). Nevertheless, ABA should have a secondary role since the levels of ABA are still increasing while the pH starts to drop in the apoplast (Zhang et al., 2016). Importantly, alkaline soils induce similar effects on plants as salt stress and both stresses have a synergistic effect exacerbating the plant responses when both are present (Xu et al., 2013), likely because the plants tend to accumulate more Na^+^ in alkaline substrates (Guo et al., 2010).

Moderate water stress enhances H^+^ efflux in the root apoplast to facilitate water uptake (Siao et al., 2020), but alkalinization of the apoplast is observed in the leaf (Geilfus, 2017). The same stimulus having an opposite effect on pH_apo_ fluctuation in different plant tissues suggests that pH_apo_ might originate the signal to the plant response but the direction of the pH_apo_ changes provides the meaning of the pH signaling.

### Biotic stress

pH_apo_ regulation during biotic interactions is time, spatial and microbial dependent, thus generalizations are not possible. Some fungal pathogens, such as the root vascular fungus *F. oxysporum* (Fo), induce fast apoplastic acidification in the first stages of contact by increasing the AHA activity, which seems to be required for plant defense (Kesten et al., 2019). Later, a strong transcriptional downregulation of the AHAs and an upregulation of their inhibitors (Menna et al., 2021), together with the secretion by Fo of RALF peptide, provokes an apoplastic alkalinization that favor Fo infection (Masachis et al., 2016). However, it remains to be explained how the fungus advances through a CW which might become stiffer upon alkalinization. Conversely, other fungal pathogens, like *Sclerotinia sclerotiorum*, induce acidification by the secretion of organic acids, which induce cell death (Cessna et al., 2000).

Microbes and small herbivores cause wounds and mechanical stress to plants. The response to these alterations includes the synthesis *in situ* of the plant hormone Jasmonic Acid (JA). JA triggers a cellular response that can travel quickly from the wound to distal cells and from the root to the shoot. In this process, the alteration of the pH_apo_ is essential to create the intracellular signaling that includes the SWPs, glutamate, Ca^2+^, and ROS response. Concretely, during wound response, the activity of AHA1 is transiently inhibited at the beginning of the signaling, alkalinizing the pH_apo_. The apoplastic proton variance is required to generate the PM depolarization and to propagate the SWP. The AHA activity is also important during the PM repolarization phase, where its activity is recovered (Kumari et al., 2019; Shao et al., 2020).

Soil pH alters the microbiota of the rhizosphere that can potentially interact with the root. In general, acid soils enhance fungal over bacterial growth, which might favor fungal pathogens spreading, and alkaline soils favor bacterial growth (Rousk et al., 2009, 2010). However, acidification of the rhizosphere has also been reported to enhance the spread of soil-borne bacterial pathogens (Li et al., 2017).

Rhizosphere and pH_apo_ proton levels influence the growth of both the plant and the intruder and their defense and virulence, respectively. Nevertheless, it is the timing and the dynamic of the pH_apo_ changes that have a leading role in biotic interactions, and the organism that controls or tolerates better those pH oscillations prevails. We propose that those pH_apo_ changes which return to basal levels, such as the strong and fast acidification seen upon Fo contact, would have the potential to contribute to cell signaling. On the other hand, stress-induced long-term pH_apo_ changes would be a structural situation as a product of the earlier signaling.

## Perspectives for the pH_apo_ role in plant biology

pH_apo_ fluxes in response to physiological needs or environmental stress might have strong consequences for plant cells beyond the ones described so far. Based on different lines of evidence discussed through this review, we predict that the intensity, duration, direction, and tissue/cellular localization of the pH_apo_ changes could act as a signal for the root to cope with those different and very challenging stresses. Therefore, in this last section, we discuss potential areas of research to undertake in the next years.

### pH microdomains within the CW

The apoplast is a heterogenous and dynamic compartment and so is its pH. Although some works have shown those differences in the pH_apo_ within different apoplastic regions (Sentenac and Grignon, 1981, 1987; Martinière et al., 2018), pH_apo_ measurements are presumably an average of different subdomains and future experiments should be focused on revealing the dynamics of those pH microdomains *in vivo*. It is particularly interesting to resolve the participation of specific components of the CW in pH_apo_ since CW has a different composition even in different areas of the same cell.

### Role of pH in the cellular cortex

An interesting concept to explore in the future is the alteration that the pH_apo_ exerts on the cell cortex pH. AHA activation, and the consequent apoplastic acidification, modify the pH on the internal side of the PM, while the rest of the cytosol is able to keep the pH stable (Kesten et al., 2019). Thus, the pH in the cell cortex area is slightly different from the rest of the cytosol and presumably should be tightly regulated to maintain the PMF (Figure 2). Moreover, variations in the cell cortex pH should alter the important cellular processes that occur in this area, such as the cortical microtubules polymerization and depolymerization, and the activation of cell surface receptors and transporters (Lomin et al., 2012; Kour et al., 2021; Lanassa Bassukas et al., 2022). However, we still need to understand this regulation.

### Interlink between plant hormones and pH_apo_

Different stresses tend to activate more than one hormonal pathway and, in some cases, the transcriptional responses from each hormone are specific to the stress (Nemhauser et al., 2006). Interestingly, one of the common points in this interplay is the AHA-dependent pH_apo_ changes, which happen concomitantly with the action of the specific plant hormone. Understanding how those phytohormone-dependent pH_apo_ changes are connected with a certain response of the plant might provide a potential biotechnological tool to enhance plant resilience under different stresses.

### New molecules to enhance plant tolerant through pH_apo_ modification

Higher pH_apo_ together with the increase in ROS, certain plant hormones, or cytosolic Ca^2+^ waves stimulate the deposition of callose, lignin, and suberin in the CW. These highly resilient molecules help to protect the cell against abiotic and biotic stresses (Miedes et al., 2014; Geilfus et al., 2017; Bacete et al., 2018; Vaahtera et al., 2019), but plant cell growth becomes seriously compromised. Nowadays, the use of microbial and plant CW-derived molecules is a potential strategy to prime plant tolerance to pathogens (Molina et al., 2021), although their impact on crop yield remains a problem to be solved. We envision that the identification of molecules that modulate the pH_apo_ in a more localized, transient, and weaker way will be an interesting biotechnological solution to promote plant stress tolerance with lower collateral effects on growth.

## Conclusion

Environment and internal signaling integration provides the plant with the proper perception of its biological status. In this sense, pH_apo_ is a ubiquitous element that emerges as a perfect candidate to unify the different inputs and generate a homogenous response. The pH_apo_ is connected with well-described cell messengers: plant hormones, ROS, and cytosolic Ca^2+^. Although many aspects remain unsettled, pH_apo_ might be the general switch that plant physiology relies on.

## Author contributions

FMG-A, CS-R, and JCM conceived the review. FMG-A and JCM wrote the review with comments and edits from CS-R. All authors contributed to the article and approved the submitted version.

## Funding

The work described in this review was supported by the Vontobel Foundation to FMG-A and CS-R, and the ETH Zurich Career Seed Grant (SEED-09 21-1) to FMG-A and JCM. Open access funding provided by ETH Zurich.

## Conflict of interest

The authors declare that the research was conducted in the absence of any commercial or financial relationships that could be construed as a potential conflict of interest.

## Publisher's note

All claims expressed in this article are solely those of the authors and do not necessarily represent those of their affiliated organizations, or those of the publisher, the editors and the reviewers. Any product that may be evaluated in this article, or claim that may be made by its manufacturer, is not guaranteed or endorsed by the publisher.
